# Extracellular electron transfer from cathode to microbes: application for biofuel production

**DOI:** 10.1186/s13068-016-0426-0

**Published:** 2016-01-19

**Authors:** Okkyoung Choi, Byoung-In Sang

**Affiliations:** Department of Chemical Engineering, Hanyang University, 222 Wangshimni-ro, Seongdong-gu, Seoul, 04763 South Korea

**Keywords:** Bioelectrochemical synthesis, Extracellular electron transfer, Cathodic electron, Electrofuel

## Abstract

Extracellular electron transfer in microorganisms has been applied for bioelectrochemical synthesis utilizing microbes to catalyze anodic and/or cathodic biochemical reactions. Anodic reactions (electron transfer from microbe to anode) are used for current production and cathodic reactions (electron transfer from cathode to microbe) have recently been applied for current consumption for valuable biochemical production. The extensively studied exoelectrogenic bacteria *Shewanella* and *Geobacter* showed that both directions for electron transfer would be possible. It was proposed that gram-positive bacteria, in the absence of cytochrome C, would accept electrons using a cascade of membrane-bound complexes such as membrane-bound Fe-S proteins, oxidoreductase, and periplasmic enzymes. Modification of the cathode with the addition of positive charged species such as chitosan or with an increase of the interfacial area using a porous three-dimensional scaffold electrode led to increased current consumption. The extracellular electron transfer from the cathode to the microbe could catalyze various bioelectrochemical reductions. Electrofermentation used electrons from the cathode as reducing power to produce more reduced compounds such as alcohols than acids, shifting the metabolic pathway. Electrofuel could be generated through artificial photosynthesis using electrical energy instead of solar energy in the process of carbon fixation.

## Background

An eventual replacement of fossil energy source with sustainable energy system is unavoidable. Biofuels have emerged as one of the sustainable fuels sources and it is considered as alternatives to petroleum. Biomass captured the energy from sunlight and stored it as high-energy chemical bonds, which is used for biofuels. More recently, electrofuels have been studied for liquid fuels as a means for intermittent electricity storage [1] using the energy of low-potential electrons such as hydrogen gas, reduced metal, or electricity [2]. It usually uses the interaction between microbes and electrode, through extracellular electron transfer.

Bioelectrochemical synthesis (BES) uses extracellular electron transfer of microorganisms catalyzing anodic and/or cathodic reactions. BES has two categories according to the direction of electron flow, microbial fuel cells (MFC, electricity production), and microbial electrosynthesis (MES, electricity consumption). A microbial fuel cell uses extracellular electron transfer to an electrode originating from organic compounds consumed by microorganisms. Microbial electrosynthesis uses electron transfer from an electrode to microorganisms producing reduced biochemical compounds. An electrode is thus used as an electron acceptor (MFC) or an electron donor (MES).

Extracellular electron transfer has been gaining wide interest in relation to microbial electrochemical synthesis [1, 3], interspecies electron transfer [4, 5], and microbial immobilization of heavy metals for bioremediation [6, 7] (Table 1). In particular, biofuels or biochemicals are reduced compounds and the reducing power is needed in microbial fermentation processes [8, 9]. An external supply of electrons using electricity enhances the reducing process in microbial metabolism. Direct electron transfer is ideal in extracellular electron transfer from a cathode to microbes.

**Table 1:** The application of bioelectrochemical reduction

Application	Product	Reaction conditions	Key outcomes	Ref.
Direct reduction	Cr^6+^ → Cr^3+^	*G. sulfurreducens*, −600 mV vs. Ag/AgCl	U(VI) was removed and recovered using poised electrode	[19]
		*Shewanella oneidensis* MR-1, −500 mV vs. Ag/AgCl	Lactate and the electrode as the electron donors for Cr(VI) reduction	[18]
	Fumarate → succinate	*G. sulfurreducens*, −500 mV vs. Ag/AgCl	Fumarate reduction dependent on current supply	[48]
		*Shewanella* species in biocathode of microbial fuel cell	Similar comparison under chromate reducing condition	[102]
	Nitrate reduction	Nitrifying and denitrifying microorganisms at +197 mV vs. SHE	Simultaneous occurrence of nitrification and denitrification at a biocathode	[49]
		Denitrifying microorganisms at −123 mV vs. SHE	Long-term stability, carbon-free operation	[51]
Indirect reduction	Caproate and caprylate production from acetate	Acetate fed at −0.9 V vs. NHE	In situ-produced hydrogen as electron donor, low concentration and reaction rates	[90]
	Ethanol production from acetate	−550 mV vs. NHE, artificial mediator tested	Methyl viologen increased ethanol production but inhibited butyrate and methane formation, still hydrogen was coproduced at the cathode	[81]
	Alcohol formation from glycerol	Open circuit operation	Changes in microbial community and product outcomes after current supply	[87]
	Reduction of acetate and butyrate to mainly alcohols and acetone	−820 mV vs. Ag/AgCl	Halotolerant mixed sulfate-reducing bacteria culture	[92]
	Polyhydroxyalkanoates (PHA) from glucose	512 mV, the biocathode coupled to a bioanode in an MEC	Microaerophilic microenvironment at cathode enhanced PHA synthesis as alternative pathway to re-oxidize the NADH	[94]
	Butyraldehyde to butanol	Immobilized alcohol dehydrogenase at −400 mV vs. Ag/AgCl	Reduction to alcohol by current without supplement of NADH	[88]
	Hydrogen production	−700 mV vs. Ag/AgCl	Increased cathodic hydrogen efficiency on microbial biocathode based on a naturally selected mixed culture	[103]
		500 mV, the biocathode coupled to a bioanode in an MEC	Operated for a long period with high current density but phosphate precipitation on the biocathode	[104]
		−700 mV vs. SHE	*Desulfovibrio* sp. as a dominant microorganism in the biocathode	[22]
	Methane production	−700 mV vs. Ag/AgCl	Methane production directly from current	[53]
		−550 mV vs. NHE	CO_2_ reduction to CH_4_, need to reduce the internal resistance	[105]
	Improved 1,3-propandiol production from glycerol	−900 mV vs. SHE	Electrical current as the driving force for a mixed population fermenting glycerol in the cathode	[93]
	Improved butanol production from glucose	+0.045 V vs. SHE	Increased alcohol production in electrofermentation with increased a ratio NADH/NAD^+^	[24]
Electrofuel from CO_2_ and electricity	Butyrate	−800 mV vs. SHE	Production of organic compounds from CO_2_ by hydrogen driven by a cathode	[100]
	Acetate	−590 mV vs. SHE	Higher acetate production than on unmodified graphite	[99]
	Acetate, 2-oxobutyrate	−400 mV vs. SHE	The production of organic acids by current consumption	[106]

The two mostly extensively studied microorganisms for extracellular electron transfer are *Geobacter* and *Shewanella* species. *Geobacter* and *Shewanella* are metal-reducing and gram-negative bacteria. Extracellular electron transfer in microorganisms is used in the metal reduction process by the microorganism and, in this case, the metal is used as an electron acceptor. When metal (hydr)oxides that are poorly soluble in water are present as electron acceptors, extracellular electron transfer occurs using multihaem c-type cytochromes in *Geobacter* and *Shewanella* [10]. Based on this phenomenon, the microorganisms are able to extracellularly transfer electrons and this can be applied for BES.

**Fig. 1:**
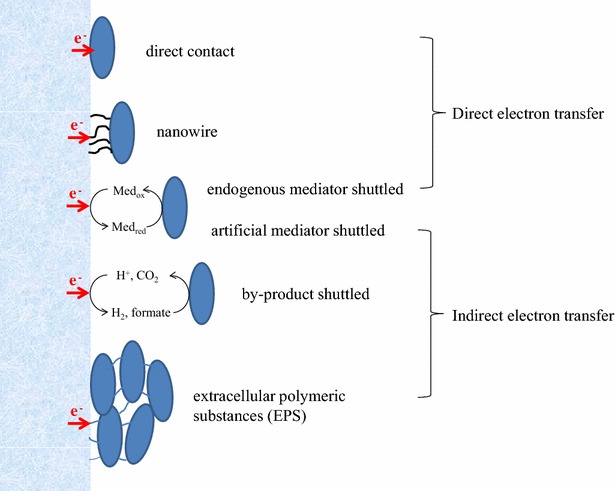
Cathodic electron transfer mode. Electrons from a cathode flow into a microbe directly, through direct contact, nanowire, and endogenous mediator; or indirectly, through an artificial mediator, by-product, or EPS

The mode of extracellular electron transfer is broadly divided into the following: (1) direct electron transfer: nanowire [11] or direct contact [12]; (2) mediators-shuttled: endogenous, exogenous as a redox compound or a by-product [13–15]; and (3) extracellular polymeric substances (EPS) of biofilms [16] (Fig. 1).

## Electron transfer from a cathode to microbes

Multihame c-type cytochrome is a key component of the electron transfer channel in gram-negative bacteria [10]. Filamentous conductive pili are also involved in electron transfer in *Shewanella* [17] and *Geobacter* [11]. BES uses two directions, i.e., microbe → electrode (anode) in MFC and electrode (cathode) → microbe in MES, with the same or different mode. Electrons flow from an electron donor with a relatively lower redox potential to an electron acceptor with higher redox potential. In this light, in the present study we address the question that of whether it is possible to use the same electron transport chain for the opposite direction.

**Fig. 2:**
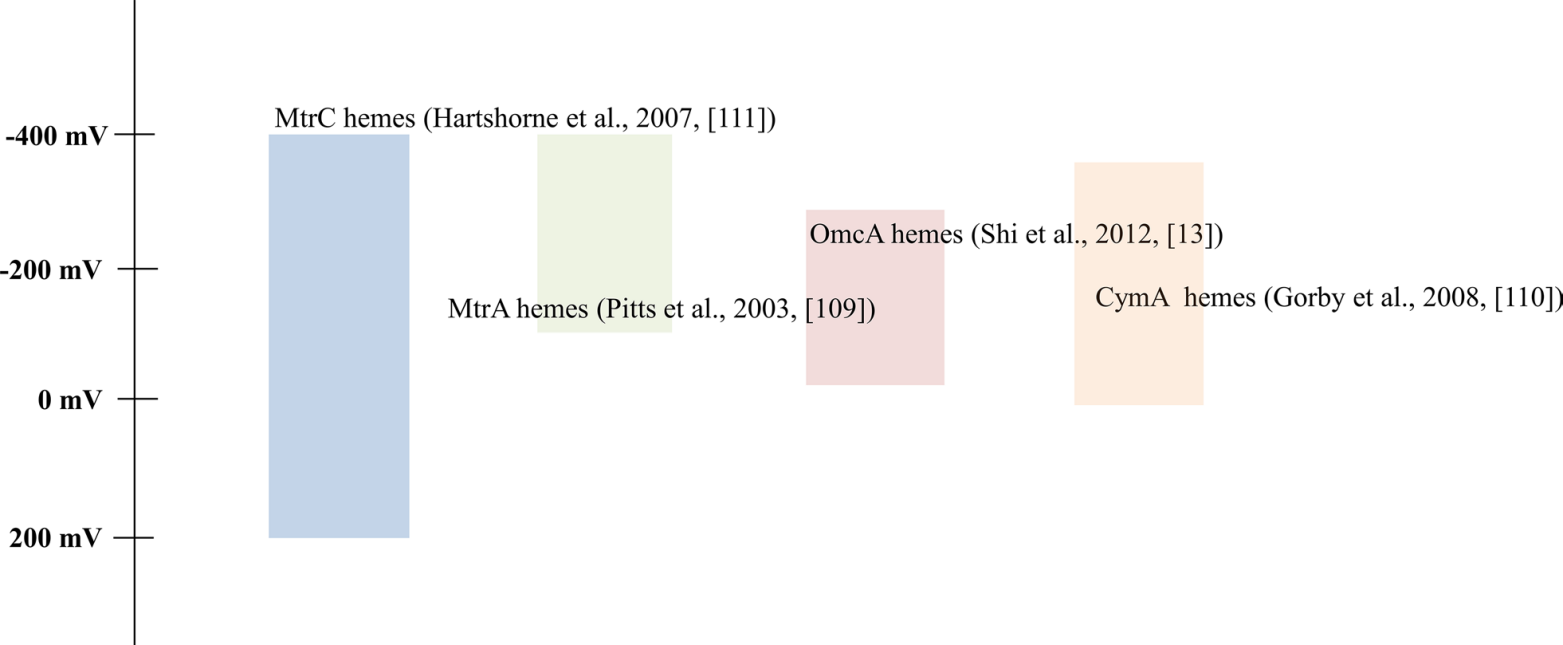
The broad range of redox potential in c-type cytochromes. Considering the possibility of bidirectional electron flow (cathodic, anodic), the broad redox potential suggests the direction of electron flow would be flexible in an electron transfer channel consisting of cytochrome C. The number in a *square bracket* represents the citation number in the reference list

The redox tower in Fig. 2 shows the broad range of redox potential for MtrC (located on an extracellular site of the outer membrane), MrtA (a periplasmic c-type cytochrome), CymA (a link point between the inner membrane and the periplasm), and OmcA (anchored in the inner membrane), which were reported to play roles in electron transfer. It is proposed that reversible electron transfer within cytochrome c complex channels is feasible and the same electron transport chain can be used for the opposite direction.

Extensively studied in MFC as iron-reducing bacteria, *Shewanella oneidensis* MR-1 [18] and *Geobacter* spp. [19] were reported to reduce the highly toxic hexavalent chromium (Cr(VI)) using a cathode. This indicates that both directions for electron transfer would be possible in current-producing bacteria, i.e., microbe → anode and cathode → microbe. However, it was reported that *Shewanella* showed a reversed Mtr pathway [20] but *Geobacter* used a different mode in the opposite direction [21].

Direct electron transfer from a cathode to microbes has been observed in a biocathode for microbial communities including betaproteobacteria [22, 23] and firmicutes [22], in addition to *Shewanella* and *Geobacter*. The presence of other electroactive, electron endergonic strains thus should be possible. *Clostridium pasteurianum* increased butanol production using cathode electron transfer without any mediator [24]. Nevertheless, the precise electron transfer channel for acceptance of extracellular electrons has not been verified; the redox enzyme in the membrane, however, may be involved in electrochemical reduction. Ferredoxin extracted from *C. pasteurianum* previously showed direct electrochemical reduction [25], but there is still no evidence of direct electron transfer through ferredoxin in whole cells. Also, several other redox proteins could be candidates for extracellular electron transfer.

## Predicted electron transfer proteins involved in extracellular electron transfer

For direct electron transfer, a membrane-bound redox protein is needed. However, there has been no study of redox proteins involved in direct electron transfer except periplasmic c-type cytochrome. Several studies have reported the possibility of direct electron transfer by microorganisms in the absence of c-type cytochrome, and here we present some possible redox proteins involved in electron transfer channels including cytochromes (Table 2).

**Table 2:** Predicted electron transfer proteins associated in extracellular electron transfer

Predicted electron transfer proteins	Active sites	Candidate microorganism associated in extracellular electron transfer	Microorganism used in bioelectrochemical system
Cytochrome C	Heme protein	Metal-reducing bacteria (*Geobacter*, *Shewanella*)	*G. sulfurreducens* [19, 48]*, Shewanella oneidensis* [18]
Ferredoxin	Fe-S protein	Clostridia, acetogens, methanogens	*Clostridium pasteurianum* [24], *Clostridium ljungdahlii, Clostridium aceticum*, *Moorella thermoacetica* [106], *Sporomusa ovata* [106, 107], *Methanothermobacter thermautotrophicus* [108], *Methanobacterium palustre, Methanococcus maripaludis* [53, 54]
Rubredoxin	Fe-S protein without acid–labile sulfur	Sulfate-reducing bacteria	*Desulfovibrio* sp. [22, 70]
Hydrogenase	[NiFe] or [FeFe] or [Fe]-only	Sulfate-reducing bacteria, methanogen	*Desulfovibrio* sp. [22, 70], methanogens [52, 53]
Formate dehydrogenase	Molybdenum or tungsten	Sulfate-reducing bacteria	*Desulfovibrio* sp. [22, 70]

### Cytochrome

The heme in cytochrome participates in electron transfer processes. Cell surface-localized cytochromes (OmcE and OmcS in *Geobacter sulfurreducens*, MtrC and OmcA in *Shewanella oneidensis* MR-1) are important components for electron transfer [26]. A microarray analysis of *G. sulfurreducens* gene transcript abundance showed the c-type cytochrome was highly expressed in current-producing biofilms [21]. However, it was suggested that the mechanism of two opposite directions, i.e., microbe → electrode and electrode → microbe, would be significantly different in *G. sulfurreducens.* Recently, cytochrome PccH with a unusually low redox potential for cytochrome (−24 mV at pH 7) located in the periplasm was proposed as a candidate to provide electron transfer in *G. sulfurreducens*, even though PccH could not be involved in the first step of accepting electrons [27, 28]. It is meanwhile known that *S. oneidensis* has a similar mechanism in both directions mainly using flavins (flavin mononucleotide and riboflavin) with cytochrome C [29].

### Ferredoxin: membrane-bound complex

Rnf complexes (a membrane-bound NADH:ferredoxin oxidoreductase) are redox-driven ion pumps and have a membrane-bound, proton-translocating ferredoxin: NAD^+^ oxidoreductase contributing to ATP synthesis (energy conservation) in acetogens such as *Clostridium ljungdahlii*. RnF is a multifunctional device with nitrogen fixation, proton translocation, and electron transport capabilities [30]. It is four flavin-containing cytoplasmatic multienzyme complexes from clostridia, acetogens, and methanogens [31] and so involved in flavin-based electron bifurcation (FBEB), which is regarded as a third mode of energy conservation in addition to substrate-level phosphorylation (SLP) and electron transport phosphorylation (ETP) [32]. Not all acetogens have rnf genes.

An energy-conserving hydrogenase (Ech) also plays a role in reducing ferredoxin with proton motive force [33]. It involves a coupling mechanism: an exergonic process attributes to coupled endergonic process; ferredoxin reduction with low potential as an exergonic reaction is coupled with H_2_ or NADH, a high-potential acceptor as endergonic reduction [34]. In methanogens, in the absence of cytochromes, methyltransferase is involved in a exergonic reaction to drive the extrusion of ions (Na^+^ or H^+^) across the membrane [35]. In the context energy conservation in a bioelectrochemical system, electron supplementation from cathode would lead to FBEB. Electron bifurcating ferredoxin reduction H^+^ gradient (for *C. ljungdahlii*) or Na^+^ (for *Acetobacterium woodii*) via membrane-bound Rnf complex was supposed as key components in electron transport chain [36].

### Rubredoxin

Rubredoxin (Rub) is also an electron transfer protein having a Fe-S cluster with relatively small molecules (about 55 amino acids) [37, 38]. It is one of the electron transfer components of sulfate-reducing bacteria (SRB) [39] and is also detected in *Clostridium pasteurianum* [37]. In *Desulfovibrio vulgaris*, Rub reduces hydrogen peroxide and superoxide [40]. Rub showed an electrochemical response with electrodes [41, 42]. Detailed roles of Rub in microorganisms have not been found but it is expected to be involved in an electron transfer channel.

### Hydrogenase and formate dehydrogenase

It was recently reported that a hydrogenase and formate dehydrogenase, which are released from cells, are adsorbed onto electrodes to accept electrons in biocorrosion and bioelectrosynthesis [43]. Methyl viologen-mediated electron transfer to hydrogenase from cathodes and mediatorless H_2_ production using cathodic electron transfer were previously suggested as electron transfer modes [44]. Formate dehydrogenase also showed direct electron transfer from cathodes [43, 45]. The periplasmic formate dehydrogenase transfers electrons to cytochrome C in *D. desulfuricans* [46]. The combination of periplasmic enzyme and c-type cytochrome likely provides the electrical wiring [44]. Several membrane-bound enzymes such as fumarate reductase [47, 48] and a denitrification enzyme [49–51] led to bioelectrochemical reduction. Therefore, a periplasmic enzyme could be involved in an electron transfer channel in bioelectrochemical systems.

## Electroactive microorganisms

### Methanogens and acetogens

The conversion of CO_2_ to CH_4_ was reported in a biocathode consisting of a methanogen via direct or indirect (H_2_ mediator) channels [52–54]. The electron donor for methanogenesis is H_2_ for autotrophic methanogens or acetate for acetoclastic methanogens. It is supposed that, as in metal-reducing bacteria, the specific electron transfer channel in methanogens plays a role in extracellular electron transfer. Abiotically produced hydrogen is also used by methanogens in indirect electromethanogenesis, instead of direct cathodic electrons [55]. While no electron transfer channel involved in electron transfer from a cathode in methanogens has been identified, energy conservation by bifurcated electron transfer in methanogens could still potentially be found [56].

The study of enzyme purification and protein identification using mass spectroscopy in an acetotrophic methanogen, *Methanosarcina acetivorans*, showed that ferredoxin reduced membrane-associated multi-heme cytochrome c in Rnf [57, 58]. Methanogens have membrane-associated hydrogenases using ferredoxin or methanophenazine as redox partners [59]. It was reported that hydrogenase and formate dehydrogenase released out of cells mediate electron transfer between a cathode and *Methanococcus maripaludis* [43]. Also, interspecies electron transfer was shown through flagellum-like appendages between *Pelotomaculum thermopropionicum* and *Methanothermobacter thermoautotrophicus* in the form of aggregates [5].

Several acetogenic bacteria (acetate production from CO_2_ and H_2_) including *Sporomusa ovata*, *Sporomusa silvacetica*, *Sporomusa sphaeroides,Clostridium ljungdahlii, Clostridium aceticum*, and *Moorella thermoacetica* consumed electrons from a cathode to reduce CO_2_ to acetate [60]. Recently, an acetogen closely related with *Sporomusa sphaeroides* was isolated and showed acetogenic growth using Fe(O) as a sole electron donor [61]. The acetogens *Moorella thermoacetica* and *C. formicoaceticum* reduced CO_2_ to formate, consuming electricity at the cathode compartment [62]. Although the mode of electron transfer to an electroactive acetogen from a cathode is still not known, the membrane-bound cytochromes and cobalt-containing corrinoids were suggested as candidates for an electron transfer channels [63]. Also, cytochrome-b enzymes (membrane-integral b-type cytochromes, −0.215 V vs. SHE) were suggested to be involved in the electron transfer process of acetogens [64].

### Metal-oxidizing bacteria and sulfur-utilizing bacteria

**Fig. 3:**
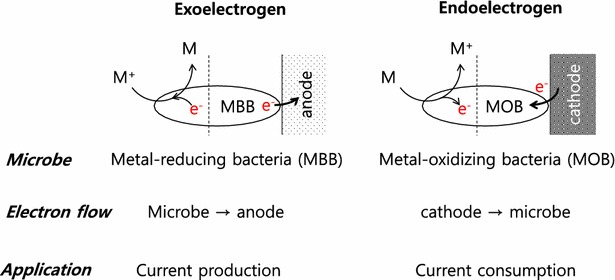
Analogy between metal-utilizing bacteria and direct extracellular electron transfer in a bioelectrochemical system. The *left side* of the *dotted line* shows the electron flow with metals in metal-utilizing bacteria and the *right side* indicates interaction with the electrode

The ability of iron-reducing bacteria to give electrons anodes gave rise to the hypothesis that iron-oxidizing bacteria (FeOB) could accept electrons from cathodes in two FeOB, *Mariprofundus ferrooxydans* and *Rhodopseudomonas palustris*, in recent studies [65–67] (Fig. 3). The marine isolate *Mariprofundus ferrooxydans* PV-1 used a cathode as a sole electron donor, generated ATP, and fixed CO_2_ [67]. *Rhodopseudomonas palustris* TIE-1 accepted electrons from a cathode, independent of photosynthesis. The dark current indicated extracellular electron uptake uncoupled from the cyclic photosynthetic apparatus and the *pioABC* operon influenced electron uptake [65]. *Rhodopseudomonas palustris* TIE-1 increased electron uptake rate 56-fold with unlimited Fe(II) supplementation in a photobioelectrochemical system [67].

It was reported that isolated marine corrosive delta proteobacterial SRB used elemental iron as the sole electron donor and reduced sulfate, showing the possibility of extracellular electron transfer [68]. Recently, a sulfide-oxidizing bacteria*, Desulfobulbaceae*, was reported to reduce oxygen in the upper layers of marine sediments using centimeter-long filaments [69]. For removal of H_2_S, the product of sulfate reduction and a toxic gas to oxygen-consuming organisms, sulfide-oxidizing bacteria used oxygen as an electron acceptor using filaments as electrical cables for H_2_S oxidation to S [69].

The SRB reduced acetate, butyrate to ethanol, butanol, respectively, using electrons through direct electron transfer from a cathode [70]. It was suggested that the direct electron transfer could take place via a redox enzyme such as cytochrome-b in SRB [70]. The SRB *Desulfopila corrodens* strain IS4 showed direct electron transfer affecting iron corrosion. Electrochemical and infrared spectroelectrochemical analyses indicated c-type cytochromes were involved in electron transfer [71]. *Acidithiobacillus ferrooxidans, Desulfosporosinus orientis,Thiobacillus denitrificans, Sulfurimonas denitrificans,* and *Desulfovibrio piger* also showed electroactivity to accept electrons from a cathode in pure cultures [72].

### Cathode modification for enhanced performance of bioelectrochemical reduction

Efforts to improve the efficiency of electron transfer between a cathode and microorganisms have focused on increasing of the interfacial area and interfacial interactions. Nanoparticle attachment on a cathode was attempted with nano-nickel [73], carbon nanotubes [74, 75], conjugated oligoelectrolytes (COEs) [76], and carbon nanotubes on reticulated vitreous carbon (NanoWeb-RVC) [74, 77]. Also, a graphene-modified biocathode enhanced bioelectrochemical production of hydrogen in a MES system [78].

Another attempt involved positively charged surface modification. Extracellular electron transfer from a cathode to a microbe was increased using a positively charged functional group on the surface of a cathode [74]. Negatively charged *S. ovate* preferred to attach on a cathode and enhanced acceptance of electrons from the cathode for the reduction of CO_2_ to acetate [74]. The positively charged anode led to an enriched biofilm on an anode but the negatively charged cathode has a repulsive interaction with microorganisms because the cell walls of most bacteria have an overall net negative charge. Therefore, attachment with microorganisms on a cathode has a charge barrier and one study showed that both the zeta potential and the hydrophobicity of cells increased in a current-consuming biofilm [24]. Modification should be tried according to the changes of cell surface characteristics on a cathode, in contrast with on an anode.

### Application for valuable biofuel production

A study of the life cycle assessment (LCA) showed MFCs do not give environmental benefit relative to the conventional anaerobic treatment [79]. The development of the MEC system connected with valuable product formation was suggested for positive energy gain [79, 80]. Thus, the product developments using bioelectrochemical reaction between microbe-cathode are promising research directions.

#### Metabolic shift to reduced compound production (electrofermentation)

**Fig. 4:**
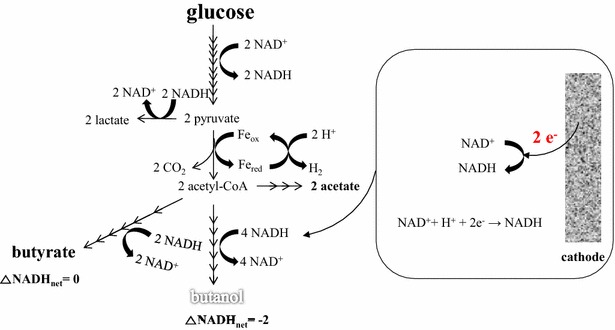
The microbial metabolic pathway of NADH-consuming compound production. One *arrow* indicates one step of reaction. The butyrate is NADH-balanced and generally produced more than butanol. The NADH reduction (inset) by electricity increases the flux of electron for butanol formation, more NADH-consuming pathway. $\varDelta {\text{NADH}}_{\text{net}} \left( {\text{per one mol of product}} \right) = {\text{NADH production}} - {\text{NADH consumption}},$ *Fe*_*ox*_ oxidized form of ferredoxin, *Fe*_*red*_ reduced form of ferredoxin

Electron transfer via an artificial mediator from a cathode has been applied in several studies and it showed an increase of reduced compound production [15, 81–85]. The distribution of final products would be determined by the electron and carbon flow in the fermentation process. Therefore, it is important to control the electron/carbon flow accordingly for production of the targeted bioproduct. Recently, an increase of butanol production in *C. pasteurianum* in a bioelectrochemical system showed the reducing power from a cathode could shift the metabolic pathway to solvent production [24]. The supplement of electrons via the cathode into a microbe led to enhanced reduction reaction directly (working on surface-associated redox enzymes, such as hydrogenases and presumably dehydrogenases [43]) or indirectly (increasing a reduced cofactor such as NADH, Fig. 4). The direct reduction process was studied in fumarate reduction to succinate [47], nitrate reduction to nitrite [48], nitrobenzene reduction to aniline [86], and hexavalent chromium reduction [18]. The indirect reduction process includes ethanol production from acetate [81], alcohol formation from glycerol [87], and butyraldehyde to butanol [88].

#### Reduction for value-added bioproducts: chain elongation

The interaction between a cathode and microbes led to reverse β oxidation [89] and reduced propionate (C3) to valerate (C5) in a glycerol-fed bioelectrochemical system [87]. Without fermentable substrates, the reduction of acetate (C2) to caproate (C6) and caprylate (C8) took place in a *Clostridium kluyveri*-predominant mixed culture in a bioelectrochemical system at −0.9 V vs. NHE cathode potential using in-situ produced hydrogen as an electron donor [90]. The reduction of acetate (C2) and butyrate (C4) into alcohols (C1 ~ C4), acetone (C2) and caproate (C6) occurred in a mixed culture of SRB at a potential of −0.85 V vs. Ag/AgCl via direct electron transfer [70].

**Fig. 5:**
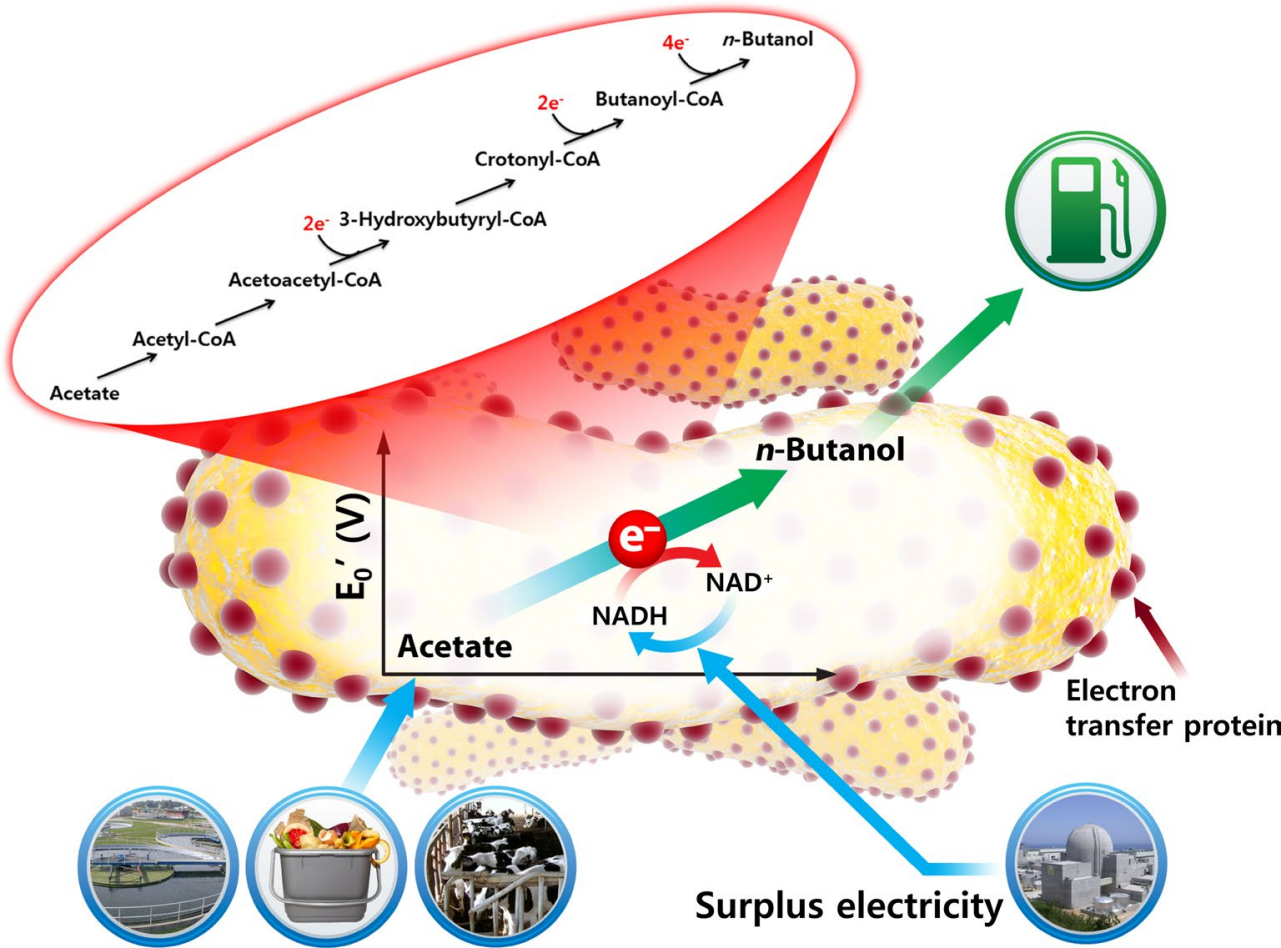
The upgrade of waste into value-added biofuels. The acetate from wastes, such as waste activated sludge, food waste, and animal manure, was feed stocks for biofuel production by electroactive microorganisms. The extracellular electron transfer from cathode to microbe via electron transfer protein could be used for the reduction of acetate to butanol. NADH the reduced form, NAD^+^ the oxidized form of nicotinamide adenine dinucleotide, respectively

The application of a cathode for additional reducing power can improve low-grade chemicals to valuable biofuels with energy supplement through the reduction process of an acid to alcohol or by chain elongation. In particular, landfill leachate, which contains acetate, propionate, and butyrate as main components [91], could be used as feed stocks in bioelectrochemical systems to upgrade waste to value-added biofuels, for examples, acetate to butanol [92] (Fig. 5), glycerol to 1,3-propandiol [93], glucose to polyhydroxyalkanoates (PHA) [92].

#### Electrofuel production (CO_2_ fixation)

Various electron sources can be used as electron donors (organic compounds, H_2_, H_2_O, etc.) or acceptors (O_2_, metal, CO_2_, etc.) by microbial organisms, whereas humans only use organic carbon as an electron donor and O_2_ as an electron acceptor. BES uses an electrode as an electron donor (cathode) or an electron acceptor (anode). In particular, electrofuel is a carbon fixation process using a cathode as an electron donor and CO_2_ as a carbon source, and this process mimics natural photosynthesis in plants [95–97].

$$n{\text{CO}}_{2} + \left( {6n{\,+\,2}} \right)\left( {e^{{{ - }1}} + {\text{H}}^{ + } } \right) \to {\text{C}}_{n} {\text{H}}_{2n + 2} + 2n{\text{H}}_{2} {\text{O}}$$

Electrofuel has several advantages: (1) the CO_2_ greenhouse gas can be used as a substrate, and the efficiency of the electricity to chemical commodities is relatively high (80 ~ 90 %), i.e., more efficient than photosynthesis; (2) the electricity can be from many renewable sources; and (3) it has good specificity to produce desired chemical commodities. However, research in this ara is an early stage and the final titer is low and the CO_2_ reduction rate is slow [98].

An acetogen used an electrode as an electron source to produce 2-oxobutyrate as well as acetate [60]. The long-term operation of a bioelectrochemical system with CO_2_ produced acetate at a level of 10.5 g/L over 20 days [99]. However, the concentration of other carbon compounds was still small, such as butyrate 35 mg/L [100], isobutanol 846 mg/L, and 3-methyl-a-butanol 570 mg/L [13].

In the absence of direct electron transfer, hydrogen led the reduction process with a hybrid microbial–water-splitting catalyst system [13, 101]. The hydrogen from water splitting was used to reduce carbon dioxide to produce liquid fuels and engineered *Ralstonia eutropha* produced isopropanol up to 216 mg/L [101]. Fermentative hydrogen production enhanced at −0.6 V vs. SHE led to increased 1,3-propandiol production [93]. Electrochemical generation of formate also mediated electron supplementation to microbes from a cathode in BES [13].

## Conclusions

**Fig. 6:**
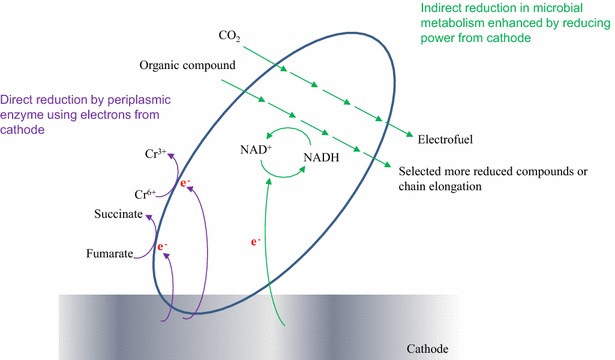
The application of bioelectrochemical reduction for cathodic electron transfer from a cathode to a microbe

The cathodic reaction in BES is of increasing concern in the context of producing alternative fuels. Beginning with metal-utilizing bacteria, several electroactive bacteria were found and applied for the conversion of electrical to chemical energy as biofuels or biotransformation (Fig. 6). Nonetheless, many technical challenges must still be addressed and the titer of final product is also low. However, research is still in an early stage and efforts such as cell membrane modification and cathode surface modification would enhance the efficiency of BES, as shown in previous studies on MFC.

## Abbreviations

BES: bioelectrochemical synthesis
COEs: conjugated oligoelectrolytes
Ech: energy-conserving hydrogenase
EPS: extracellular polymeric substances
ETP: electron transport phosphorylation
FBEB: flavin-based electron bifurcation
FeOB: iron-oxidizing bacteria
MES: microbial electrosynthesis
MFC: microbial fuel cells
Rub: Rubredoxin
RVC: reticulated vitreous carbon
SHE: standard hydrogen electrode
SLP: substrate-level phosphorylation
SRB: sulfate-reducing bacteria
